# Nutritional Education Through Internet-Delivered Menu Plans Among Adults With Type 2 Diabetes Mellitus: Pilot Study

**DOI:** 10.2196/resprot.2525

**Published:** 2013-10-11

**Authors:** Abeer Bader, Réjeanne Gougeon, Lawrence Joseph, Deborah Da Costa, Kaberi Dasgupta

**Affiliations:** ^1^School of Dietetics and Human NutritionMcGill UniversityMontreal, QCCanada; ^2^Division of Clinical EpidemiologyDepartment of MedicineMcGill UniversityMontreal, QCCanada

**Keywords:** weight loss, obesity, hemoglobin A1C, blood pressure, Internet, Web, type 2 diabetes mellitus, diet, menu

## Abstract

**Background:**

A potential barrier to weight loss and vascular risk reduction is difficulty in operationalizing dietary education into a concrete plan. Although a variety of Internet-based software tools are now available to address this issue, there has been little formal evaluation of these tools.

**Objective:**

The aim of this single-arm pilot study is to determine the effect of a 24-week Internet-based menu-planning program, by examining pre- to postintervention changes in the body weight, blood pressure, and glycemia, specifically among overweight adults with type 2 diabetes mellitus (DM2), a clinical population at high risk for vascular diseases.

**Methods:**

A total of 33 adults with DM2 were recruited by collaborating registered dietitians to a 24-week Internet-based menu-planning program. Individualized dietary prescriptions were operationalized into weekly Internet-delivered menu plans through an adapted version of a commercially available service. Adherence was defined as logging into the program at least once per week for a minimum of 18 of the 24 weeks. Multiple imputations were used for missing data. Using baseline and postintervention assessments, we calculated the weight changes (mean, 95% CI) and investigated the corresponding effects (linear regression models) on blood pressure (systolic, diastolic) and hemoglobin A1C (ie, glycemia).

**Results:**

The mean age was 58 (SD 7) years and the mean baseline body mass index was 34.4 (SD 4.6) kg/m^2^. The results of this study showed that ≥5% weight reduction was achieved by 6/33 participants (18%) and by 5/18 adherent participants (28%). A mean weight change of −2.0% (95% CI −2.6 to −1.4) was observed, with changes occurring in the adherent (−3.6%, 95% CI −4.5 to −2.8) but not in the nonadherent (0%, 95% CI −0.6 to 0.7). It was found that each 1% reduction in body weight was associated with a −2.4 mmHg change in systolic (95% CI −3.5 to −1.2) and a −0.8 mmHg change in diastolic blood pressure (95% CI −1.4 to −0.2). Percent weight change was not found to be related to changes in A1C.

**Conclusions:**

In adults with DM2, an Internet-based menu-planning program has the potential to lead to clinically important weight reductions in more than one quarter of those who adhere, with corresponding improvements in blood pressure.

## Introduction

Although many adults with type 2 diabetes mellitus (DM2) tend to be overweight, loss of 5% of total body weight leads to improvements in vascular risk factors [1,2] and loss of 2-5% of weight may confer benefits [3]. Difficulty in translation of dietary education and advice into a concrete operational plan (ie, grocery lists, recipe selection, time management, and budgeting) is a potential weight loss barrier. Indeed, assistance with menu planning has been demonstrated to be effective in overweight individuals to achieve weight loss [4]. Unfortunately, because of time constraints and client volume, clinicians are generally unable to provide daily meal plans and recipes.

Several investigators have recently attempted to circumvent the menu-planning barrier through the use of prepared meals. This approach has been demonstrated to be highly effective in realizing the benefits associated with weight loss. For example, in a clinical trial conducted among overweight women, weight losses were greater among participants who received free Jenny Craig prepared meals (n=167; 42-67% of their total energy intake), with a net 10.9% loss compared to a 2.6% loss in the control group (n=111) who followed dietary guidelines [5]. More than 60% of the intervention arm participants achieved a≥5% weight loss. Others have opted to examine the use of meal replacements in the form of shakes and bars, which is potentially a less expensive option. Impressively, in adults with DM2, the Look AHEAD trial incorporated meal replacements to achieve a 7% or greater weight loss in 1 year, and the mean reduction in the intervention arm (n=2570) was 8.6% [6].

An ongoing reliance on prepared meals and/or meal replacements, however, may not be financially realistic or appealing to some individuals. Such individuals may benefit from menu-planning services delivered through the Internet. These plans may be generated through specialized software that integrates “dietary prescriptions” with banks of recipes and food items locally available. However, while the Internet is now emerging as a source of many menu-planning tools, there has been little formal evaluation of their effectiveness. An Internet-based menu-planning strategy, known as eDiets, has been previously implemented in overweight individuals [7,8]. Among completers (n=48) of the eDiets study of Gold and colleagues, a weight loss of 5% or greater was seen in 18 (37%) of the participants. No previous studies to our knowledge have been conducted in overweight persons with DM2. We report herein the results of our 24-week intervention in overweight adults with DM2 who received dietary education from a registered dietitian and were then given weekly individualized menu plans via the Internet, through an adapted version of an existing program [9]. We evaluated pre- and postintervention changes in weight and dietary intake, as well as the corresponding effects of weight change on glycemic control and blood pressure.

## Methods

### Design

We conducted a single-arm pilot interventional study to determine the effect of a 24-week Internet-based menu-planning program by examining pre- to postintervention changes. This design permits each participant to act as his or her own control, to potentially reduce confounding and increase the precision of estimates in smaller pilot studies. In contrast, small randomized controlled trials (RCTs) risk unbalanced treatment arms and limited scope for statistical adjustments. The McGill Faculty of Medicine Institutional Review Board (Montreal, Canada) approved all study procedures, as did the participating institutions.

### Participants

Recruitment of participants and data collection occurred over a 52-week period (ie, from June 2009 to June 2010). Potential candidates were identified by registered dietitians working in diabetes outpatient clinics in Montreal, affiliated with McGill University (McGill University Health Centre, Jewish General Hospital, and St. Mary’s Hospital Center). Dietitians invited all potentially eligible patients to participate. Those who indicated interest were referred to the study coordinator. To be eligible to participate in the study, participants were required to meet the following inclusion criteria: diagnosis of DM2, a body mass index of 25 to 45 kg/m^2^, and regular access to computer and Internet services. Participants were deemed ineligible if they met any of the following exclusion criteria: history of any significant comorbid illness (eg, malignancy, renal failure, or liver disease), taking medications (eg, orlistat, steroids) that could affect weight, smoking during the last 12 months, or pregnant or planning to become pregnant within the next 12 months.

### Intervention

We tested an adapted version of a commercially available Internet-based menu program (SOSCuisine; Multimedia Appendix 1). The program offers Internet-based menu-planning services, with some services free of cost (eg, five dinner menus weekly) and others paid (eg, complete menu plan each day). In the present study, a website, without third-party advertisements, was specifically developed for evaluation purposes. Costs of the services were covered through a research grant that incurred no costs to participants.

Following informed consent and baseline assessment by research personnel, the collaborating referring dietitians shared their assessments and recommendations with the SOSCusine dietitian through telephone discussion. The measures included for the discussion were diabetes history, socioeconomic status, medication use, usual dietary intake, food habits, weight history, energy requirement for weight loss, sample meal pattern, and macronutrient distribution. The nutritional requirements were added on SOSCuisine software to develop an individualized menu plan. The software allowed alignment of the “dietary prescription” with the SOSCuisine menu bank (over 62,000 recipes) as well as weekly specials from local grocery stores in Montreal and the number of individuals within the household. Participants received weekly menu plans, recipes, a grocery list, ingredients’ cost with and without grocery store specials, and a step-by-step action plan to reduce meal preparation time [9].

Macronutrient distribution for the management of diabetes among overweight adults followed 2008 Canadian Diabetes Association guidelines (eg, carbohydrate intake accounted for 45-60% of total energy intake, protein for 15-20%, and fat for less than 35%) [10]. Other nutritional considerations included decreased sodium intake, increased whole grain intake, including an intake of 25-50 g of dietary fiber per day, and restricted saturated fat intake to less than 7% of total daily energy intake. A detailed nutritional facts table and servings from each food group based on Eating Well With Canada’s Food Guide [10] were generated for each meal.

### Assessments

Assessments were completed at baseline (week 0) and following the intervention (ie, at 25-26 weeks) by research personnel at the Division of General Internal Medicine, Montreal General Hospital site, McGill University Health Centre. Demographic data including sex, marital status, occupation, place of birth, ethnicity, education, and income, were obtained at the baseline assessment. Baseline and follow-up measures included body weight (postvoid in light clothes without shoes to nearest 0.1 kg, using a SECA 882 electronic scale), height (head in Frankfurt horizontal plane position to nearest 0.1 cm, without shoes using a SECA 214 stadiometer), waist circumference (standing position, midway between the lateral lower ribs and the iliac crests after a moderate expiration), hip circumference (widest level, over the greater trochanters), and blood pressure (following 5-minute rest, two measures 2 minutes apart, using an Omron HEM-747 IC). Venous blood was sampled for A1C measurements (BioRad Variant II high-performance liquid chromatography system). A1C level reflects an overall glucose control during the previous 2- to 3-month period, with a target AIC level of approximately 7% in DM2. Prescription medications were recorded.

Dietary intake was estimated using a validated food frequency questionnaire [11]. Physical activity was measured using a validated self-administered International Physical Activity Questionnaire (IPAQ)—short form [12] that assesses physical activity for the prior 7 days. Participants’ stage of change was assessed using the Weight Stages of Change—short form [13]. The usage of the website usage was tracked electronically.

### Statistical Analyses

Descriptive statistics at baseline were presented as means and SDs for all continuous variables and proportions for categorical variables. These statistics were generated both for the cohort overall and stratified by adherence. Adherence in this study was defined as having logged in at least once per week for 18 of the 24 weeks (ie, 75% of total weeks). Implausible dietary intake values were excluded (ie, outside the range of 500-3500 kcal/d for women and 800-4000 kcal/d for men [14]), as were implausible physical activity values, in accordance with the IPAQ scoring protocol. Mean changes and 95% CIs were calculated for change in weight and other anthropometric measurements, such as clinical parameters, changes in dietary food intake, and changes in physical activity in MET-min/week, both overall and stratified by adherence.

Linear regression models were constructed to estimate whether reduced weight was associated with changes in systolic and diastolic blood pressure, and A1C. Multiple imputation was used to adjust for missing data [15]. Because of sample size constraints, a maximum of three variables could be included concurrently in our adjusted models. In all cases, we present age- and sex-adjusted models. For systolic and diastolic blood pressure, separate adjustments for season of baseline assessment and change in physical activity were included in our analyses. Data were analyzed using SAS version 9.2.

## Results

### General Results

**Figure 1:**
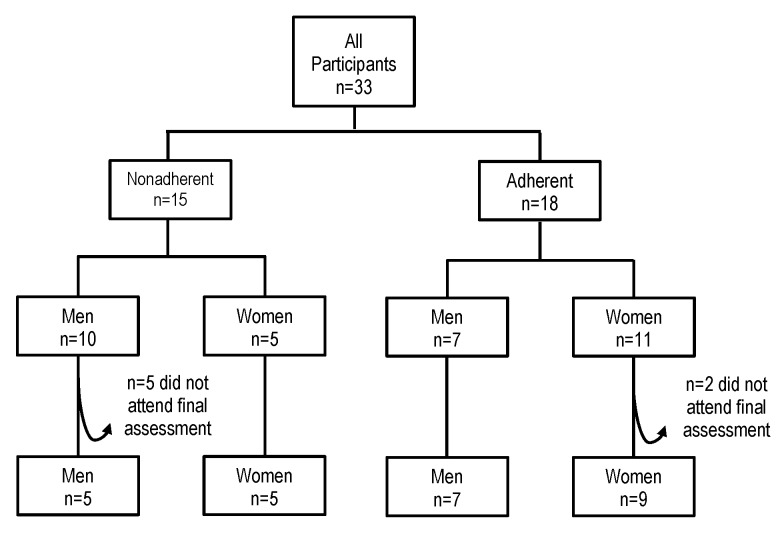
Flow diagram of the participants from enrollment to final assessment.

Prospective participants’ recruitment and data collection occurred over a 52-week period (ie, from June 2009 to June 2010). A total of 33 participants were enrolled, and 26 (79%) completed final assessments (Figure 1).

Participants recruited for the study were middle-aged to elderly with a mean age of 57.8 (7.4) years. Of all the participants, 16/33 participants (49%) were women (Table 1). Participants were predominantly married, Europid, and educated beyond high school graduation. A total of 14/33 participants (42%) were retired or not seeking work. They were on average in obese class 1 level, with elevated waist circumference and waist-to-hip ratio. Most participants were in the “action” phase of the weight-loss stage of change at baseline, and reported confidence in their ability to prepare meals. Baseline average daily energy intake was found to be approximately 2070 kcals. Of this energy intake, 100 g (19.4%) came from protein, 91 g (39.8%) from fat, including 28 g (12%) from saturated fat, and 211 g (40.8%) from carbohydrate. The mean sodium intake of 3.1 g/d (1.6 g) was also found to be above recommendations, while mean dietary fiber intake of 21.6 g/d (10.7 g) was slightly lower than daily recommendations of 25-50 g/d [10]. Participants were classified as being mainly low or moderately active. Average duration of diabetes was close to 8 years. Mean A1C and blood pressure values were slightly higher than recommended targets [10]. Antihypertensive, antihyperglycemic, and lipid-lowering medications were commonly used by participants (75.8%, 97.0%, and 81.8%, respectively).

**Table 1:** Baseline characteristics, both overall and stratified by adherence.

Variables		Total (N=33)	Nonadherent (n=15)	Adherent (n=18)
Age in years, mean (SD)		57.8 (7.4)	55.7 (8.5)	59.6 (6.1)
Women, n (%)		16 (48)	5 (33)	11 (61)
Marital status (single), n (%)		6 (18)	4 (27)	2 (11)
Ethnicity (Europid), n (%)		27 (82)	11 (73)	16 (89)
Education (high school or less), n (%)		11 (33)	0 (33)	6 (33)
Occupational status (retired or not seeking work), n (%)		14 (42)	4 (27)	10 (56)
Weight in kg, mean (SD)		95.5 (14.3)	94.3 (9.7)	96.5 (17.5)
BMI in kg/m^2^, mean (SD)		34.4 (4.6)	33.3 (4.2)	35.3 (4.8)
**Waist in cm, mean (SD)**				
	Women	103.3 (8.4)	103.0 (8.0)	103.4 (9.0)
	Men	110.5 (7.6)	108.3 (5.5)	113.7 (9.5)
**WHR, mean (SD)**				
	Women	0.85 (0.05)	0.85 (0.06)	0.85 (0.05)
	Men	0.99 (0.05)	1.0 (0.04)	0.99 (0.06)
**Weight—stage of change, n (%)**				
	Precontemplation	0 (0)	0 (0)	0 (0)
	Contemplation	5 (15)	3 (20)	2 (11)
	Action	23 (70)	10 (67)	13 (72)
	Maintenance	5 (15)	2 (13)	3 (17)
**Cooking skills, n (%)**				
	Prepares simple meals	6 (18)	3 (20)	3 (17)
	Cooks with a recipe	6 (18)	4 (27)	2 (11)
	Competent cook	17 (52)	5 (33)	12 (67)
	Expert cook	3 (9)	2 (13)	1 (6)
	Not applicable (as it is not my role to cook)	1 (3)	1 (7)	0 (0)
**Dietary intake in grams/day, mean (SD)**				
	Energy^a^	2071 (699)	2022 (503)	2112 (829)
	Protein	100.2 (35.5)	100.4 (30.9)	100.2 (39.1)
	Carbohydrate	210.8 (81.9)	219.8 (76.8)	201.1 (85.4)
	Fat	90.6 (40.6)	83.2 (34.9)	96.7 (44.0)
	Fiber	21.6 (10.7)	23.2 (9.4)	20.3 (11.6)
	Sodium	3.1 (1.6)	2.7 (1.0)	3.4 (1.9)
	Saturated fat	27.8 (13.3)	28.0 (14.2)	27.6 (12.6)
**Physical activity, n (%)** ^b^				
	Low	11 (37)	7 (47)	4 (27)
	Moderate	12 (40)	5 (33)	7 (47)
	High	7 (23)	3 (20)	4 (27)
DM2 duration in years, mean (SD)		7.6 (6.1)	7.0 (5.2)	8.0 (7.0)
A1C (%), mean (SD)		8.1 (1.5)	8.6 (1.8)	7.7 (1.1)
**Blood pressure in mmHg, mean (SD)**				
	Systolic	137 (14)	138 (11)	136 (16)
	Diastolic	83 (8)	82 (8)	84 (9)

^a^Energy intake in kilocalories/day.

^b^Adherence is defined as logging into the Internet-based menu program at least once per week for a minimum of 18 weeks of the 24-week intervention (ie, 75% of weeks).

### Evaluation Outcomes

Of the total 33 participants, 18 (54%) of them were adherent (Figure 1) by our definition. On average, participants logged into the Internet-based menu program at least once per week for 14.7 weeks (9.9 weeks). Among the adherent group (Table 1), a higher proportion of participants were women, Europid, retired, and reported being a competent cook, were in the action stage of change, and reported a high level of physical activity.

Participants’ change in weight at 24 weeks ranged from −12.4% to +4.0%, with a mean change of −2.0% (95% CI −2.6 to −1.4) overall and −3.6% (95% CI −4.4 to −2.2) in the adherent group (Table 2). A 5% or greater weight reduction was achieved by 6/33 participants (18%) overall and 5/18 participants (27.8%) in the adherent group. Only 1/33 participants (3%) overall and 1/18 adherent participants (6%) achieved a weight reduction of ≥10%. This one participant was successful in achieving a 12.4% net weight loss.

A reduction in A1C levels was found among participants (A1C change −0.4%, 95% CI −0.6 to −0.2), with the significant reduction observed in the nonadherent group. The A1C changes were not related to weight changes. It was found that excluding the 7 participants with changes in antihyperglycemic medications during the study period did not alter the direction of overall findings (ie, A1C change −0.3%, 95% CI −0.9 to 0.3).

Reductions in systolic and diastolic blood pressure were also observed (systolic change −2.1 mmHg, 95% CI −4.3 to 0.2; diastolic change −0.6 mmHg, 95% CI −1.7 to 0.5), with the adherent demonstrating a systolic change of −6.1 mmHg (95% CI −9.3 to −2.8) and diastolic change of −1.7 mmHg (95% CI −3.5 to 0.1 mmHg). There were reductions in dietary intakes of total energy, protein, carbohydrate, fat, saturated fat, fiber, and sodium. Improvements in physical activity were observed at 24 weeks, with a mean increase of 319 MET-min/week (95% CI −53 to 690).

The relationship between changes in weight and blood pressure is shown in Table 3. After adjustment for age and sex, the change of 1 kg unit for a weight (equivalent to a 1% weight change) was associated with a systolic blood pressure change of −2.2 mmHg (95% CI −3.1 to −0.6) and a diastolic blood pressure change of −0.8 mmHg (95% CI −1.4 to −0.1). It was also found that excluding the 6 patients with changes in antihypertensive medications did not alter findings. There were no clear relationships between modifications in carbohydrate intake and A1C change or between reductions in sodium intake and change in blood pressure. An increase of 100 MET-min/week resulted in −0.2 mmHg (95% CI −0.3 to −0.1) change in systolic blood pressure and −0.1 mmHg (95% CI −0.1 to −0.02) in diastolic blood pressure. Changes in physical activity did not appear to be a predictor of change in weight or A1C level.

**Table 2:** Changes overall and stratified by adherence and sex.

		Mean (95% CI)		
Variables		Total (N=33)	Nonadherent (n=15)	Adherent (n=18)
Weight loss of ≥5%, n (%)		6 (18)	1 (7)	5 (28)
Weight (kg)		−2.0 (−2.6 to −1.4)	0 (−0.6 to 0.6)	−3.6 (−4.4 to −2.8)
% Weight		−2.0 (−2.6 to −1.4)	0 (−0.6 to 0.7)	−3.6 (−4.5 to −2.8)
BMI^a^ (kg/m^2^)		−0.7 (−0.9 to −0.5)	0.1 (−0.2 to 0.3)	−1.3 (−1.6 to −1.0)
**Waist (cm)**				
	All	−2.2 (−3.0 to −1.4)	−1.7 (−2.4 to −0.9)	−2.7 (−3.9 to −1.4)
	Women	−2.6 (−3.3 to −2.0)	−0.58 (−1.6 to −0.5)	−2.3 (−4.3 to −0.2)
	Men	−1.7 (−3.2 to −0.3)	−2.0 (−3.2 to −1.2)	−3.3 (−4.1 to −2.4)
**Hip (cm)**				
	All	−2.9 (−3.6 to −2.1)	−1.4 (−2.4 to −0.4)	−4.1 (−5.1 to −3.1)
	Women	−4.1 (−5.4 to −2.8)	−1.9 (−4.6 to 0.8)	−5.1 (−6.6 to −3.6)
	Men	−1.7 (−2.3 to −1.1)	−1.1 (−1.9 to −0.3)	−2.6 (−3.5 to −1.7)
**WHR** ^a^				
	All	0 (−0.01 to 0.01)	0 (−0.02 to 0)	0.01 (−0.01 to 0.02)
	Women	0.01 (0 to 0.02)	0.01 (−0.01 to 0.02)	0.01 (0 to 0.03)
	Men	−0.01 (−0.02 to −0.07)	−0.02 (−0.03 to −0.01)	−0.01 (−0.02 to 0)
A1C (%)		−0.4 (−0.6 to −0.2)	−0.7 (−1.0 to −0.3)	−0.2 (−0.5 to 0.1)
**Blood pressure (mmHg)**				
	Systolic	−2.1 (−4.3 to 0.2)	2.7 (−0.1 to 5.5)	−6.1 (−9.3 to −2.8)
	Diastolic	−0.6 (−1.7 to 0.5)	0.9 (−0.4 to 2.2)	−1.8 (−3.5 to −0.1)
Physical activity^b^ (MET-min/week)		319 (−53 to 690)	−407 (−845 to 30)	924 (371 to 1476)
Energy intake		−418 (−518 to −318)	−463 (−584 to −343)	−380 (−536 to −224)
**Dietary intake (grams/day)**				
	Protein	−22.7 (−27.9 to −17.5)	−27.2 (−32.7 to −21.7)	−19.0 (−27.4 to −10.6)
	Carbohydrate	−38.0 (−51.4 to −24.6)	−54.9 (−74.2 to −35.6)	−23.9 (−42.2 to −5.6)
	Fat	−20.2 (−25.6 to −14.7)	−20.1 (−27.3 to −12.9)	−20.2 (−28.3 to −12.1)
	Fiber	−3.9 (−5.6 to −2.2)	−6.8 (−9.0 to −4.7)	−1.5 (−3.9 to 1.0)
	Sodium	−0.7 (−0.9 to −0.5)	−0.6 (−0.8 to −0.4)	−0.7 (−1.0 to −0.4)
	Saturated fat	−7.9 (−9.6 to −6.1)	−9.1 (−11.6 to −6.5)	−9.8 (−9.3 to −4.4)

^a^BMI, body mass index (calculated as weight in kilograms divided by height in meters squared); WHR, waist-to-hip ratio (calculated as waist circumference divided by hip circumference.

^b^Adherence is defined as logging into the Internet-based menu program at least once per week for a minimum of 18 weeks of the 24-week intervention (ie, 75% of weeks).

**Table 3:** Linear regression models examining relationships between weight change and changes in blood pressure and hemoglobin A1C.

Model		Change in outcome variable of interest per 1 kg unit decrease in weight	95% CI
**Systolic (mmHg)**			
	Weight change	−2.30	−3.50 to −1.11
	Weight change, age, sex	−2.20	−3.14 to −0.61
	Weight change, baseline season	−2.17	−3.41 to −0.93
	Weight change, change in PA	−2.17	−3.27 to −1.08
**Diastolic (mmHg)**			
	Weight change	−0.76	−1.40 to −0.13
	Weight change, age, sex	−0.75	−1.41 to −0.10
	Weight change, baseline season	−0.76	−1.43 to −0.10
	Weight change, change in PA	−0.72	−1.35 to −0.10
**A1C (%)**			
	Weight change	0	−0.14 to 0.15
	Weight change, age, sex	0	−0.15 to 0.15

## Discussion

### Principal Results

In a middle-aged to elderly cohort of adults with DM2 who consulted a registered dietitian, we determined that a 24-week program of Internet-based menu planning led to a 5% or more net weight reduction in approximately one fifth (6/33, 18%) of those who enrolled and over one fourth (5/18, 28%) of those who logged on weekly (mean 2% net weight reduction overall, 3.6% in adherent). Overall energy intake decreased and physical activity increased. There was an overall A1C reduction in this cohort (−0.4%), although it was not related to weight change. In contrast, there were significant reductions in blood pressure in the adherent group (systolic −6.1 mmHg; diastolic −1.8 mmHg); such a reduction, if sustained, is sufficient to lower the risk of future vascular complications. Blood pressure reductions overall were related to reductions in weight and increases in physical activity. These findings provide some evidence for potential effectiveness of an Internet-based menu-planning strategy (weekly plans, grocery lists, menus, and recipes) when the treating dietitian is involved in the structure of the plan, as was the case in our study. Conducting an RCT appears to be justified, based on our results.

### Comparison With Prior Work

As noted previously, two other groups of investigators have examined the effects of an Internet-based menu-planning strategy, “eDiets program” [7,8]. The weight change observed in the eDiets Internet-based menu-planning program was greater than that observed in our pilot study: among completers (n=48) of the eDiets study of Gold and colleagues, a weight loss of 5% or more was seen in 18 (37%) of the participants. However, the eDiets population included overweight individuals rather than overweight individuals with DM2. A previous study by Wing and colleagues suggests that individuals with DM2 have greater difficulty losing weight: weight loss was lower in overweight persons with DM2 compared to their overweight spouses who followed the same diet or exercise program [16]. In more recent studies, Wing and colleagues have achieved much greater weight losses in individuals with DM2 through a dietary intervention that incorporates meal replacements and strong behavioral therapy elements [17]. However, not all individuals with DM2 may be willing to use meal replacements. Therefore, for these individuals, an Internet-delivered menu plan may be a useful option, based on our findings.

Food frequency information suggested that our participants induced a caloric deficit of 418 kcal/day. Although such a deficit might be expected to result in a weight loss of 8.6 kg over a 24-week period, the actual weight loss observed was lower than this. Following the intervention, energy from fat and saturated fat intake remained 3.8% and 4.0% above recommendations for DM2, respectively [10]. Incorporating complex and low-glycemic index carbohydrates and dietary fiber is associated with lower prevalence of cardiovascular diseases and better glycemic control [10,18,19]. Overall, mean carbohydrate intake of participants at baseline was lower than recommendations and decreased by 3.9 g at final assessment.

The reduction in sodium intake was found to be 0.7 g/d overall. Sodium intake at final assessment was 2.3 g/d, which is an amount associated with reduced blood pressure in previous studies using DASH menus [20]. A relationship between the reduction in sodium intake and change in blood pressure was not established, however, in our study, perhaps because of sample size limitations.

Overall, clinically important changes in A1C were observed but reductions were more pronounced in nonadherent participants. Notably, however, baseline A1C values were roughly 1.0% greater among nonadherent participants compared to those who adhered (Table 1); this could have contributed to the greater A1C change noted in the nonadherent group (ie, greater “room” to decline).

Further, we observed reductions in blood pressure among adherent participants. Blood pressure changes corresponded to weight changes. In our study, a 1 kg loss of body weight was associated with 2.2 mmHg decrease in systolic blood pressure (95% CI −3.1 to −0.6); thus, a 10 kg reduction might be extrapolated to lead to a 20 mmHg decrease. This relationship between weight change and systolic blood pressure change is in agreement with what would be expected from previous studies [21]. In our study, increased physical activity was associated with improvements in both systolic and diastolic blood pressure. These results are also consistent with previous literature [21]. We would note that while participants were encouraged to engage in regular physical activity by their dietitians and on the website, this was not a specific focus of the intervention.

### Limitations

The limitation of our study is its small sample size, but, as noted above, precise estimates (ie, narrow CIs) of effect measures were obtained. Due to funding limitations, the study involved comparisons of pre- and postintervention values rather than a randomized controlled design with an intervention and control group. However, we would note that we endeavored to link the program with changes by assessing the relationship between weight changes and changes in glycemia and blood pressure. With respect to the intervention itself, it may have been strengthened by greater emphasis on self-monitoring [7,8,22,23] and motivational messages to increase the frequency of logging into the program and to improve patient adherence to dietary recommendations. We opted not to further modify the existing program because we wanted to evaluate a “real-life” program, as such a program would potentially be accessible for use even after the study. At the time of the final evaluation, several participants endorsed the utility of the Internet-based tools; however, timelines and funding precluded a systematic qualitative assessment. We acknowledge this as a limitation, and note that we have performed such assessments in other examinations of behavioral interventions [24,25].

### Conclusions

Our pilot study findings indicate that in adults with DM2, recruited from standard care clinics, nutritional prescriptions operationalized through an Internet-delivered menu-planning strategy may improve the vascular risk profile of an important proportion of participants who log in regularly. This appears to occur through weight changes that lead to blood pressure reductions, even in the context of antihypertensive therapy. The effects on glycemic control, however, are not clear. The strategy could be strengthened through greater emphasis on self-monitoring and motivational support.

Internet-based tools have the advantage of ease of access to a large number of individuals at their own convenience. Our study adds to the evidence base that this type of strategy may be a modern adjunct to diabetes care in those with Internet access who log in regularly. Behavioral nutrition approaches may be conceptualized as ranging from less structured to more structured approaches. These range from gaining familiarity with general nutritional principles, acquiring a planning framework (eg, carbohydrate exchanges, “points”), receiving plans, and planning tools to using meal replacements or prepared meals. Internet-based tools lie in the midrange of this spectrum, providing plans and tools, but still allowing consumption of home-cooked meals. In combination with reliable dietary education, our study suggests that these tools may have some beneficial effects. Our findings may be used to inform an RCT to definitively test this possibility.

## Multimedia Appendix 1

Sample of a 1-day diabetes menu plan.

## Abbreviations

DM2: type 2 diabetes mellitus
IPAQ: International Physical Activity Questionnaire
RCT: randomized controlled trial
