# Supplementary material for: Nutritional Education Through Internet-Delivered Menu Plans Among Adults With Type 2 Diabetes Mellitus: Pilot Study
Source: JMIR Res Protoc. 2013 Oct 11;2(2):e41. doi: 10.2196/resprot.2525 (PMC3806354; doi:10.2196/resprot.2525)

## Slide 1
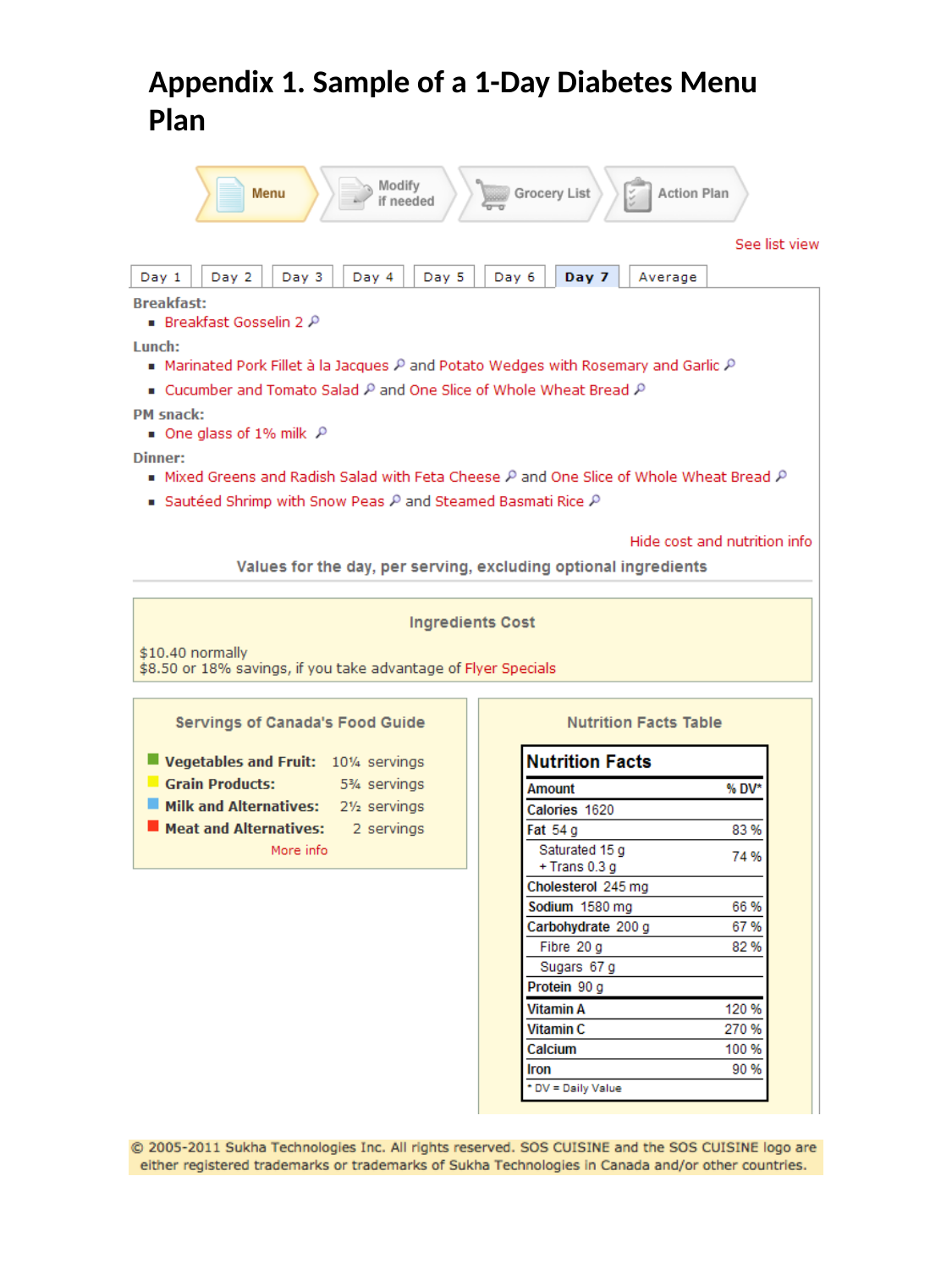

Appendix 1. Sample of a 1-Day Diabetes Menu Plan

## Slide 2
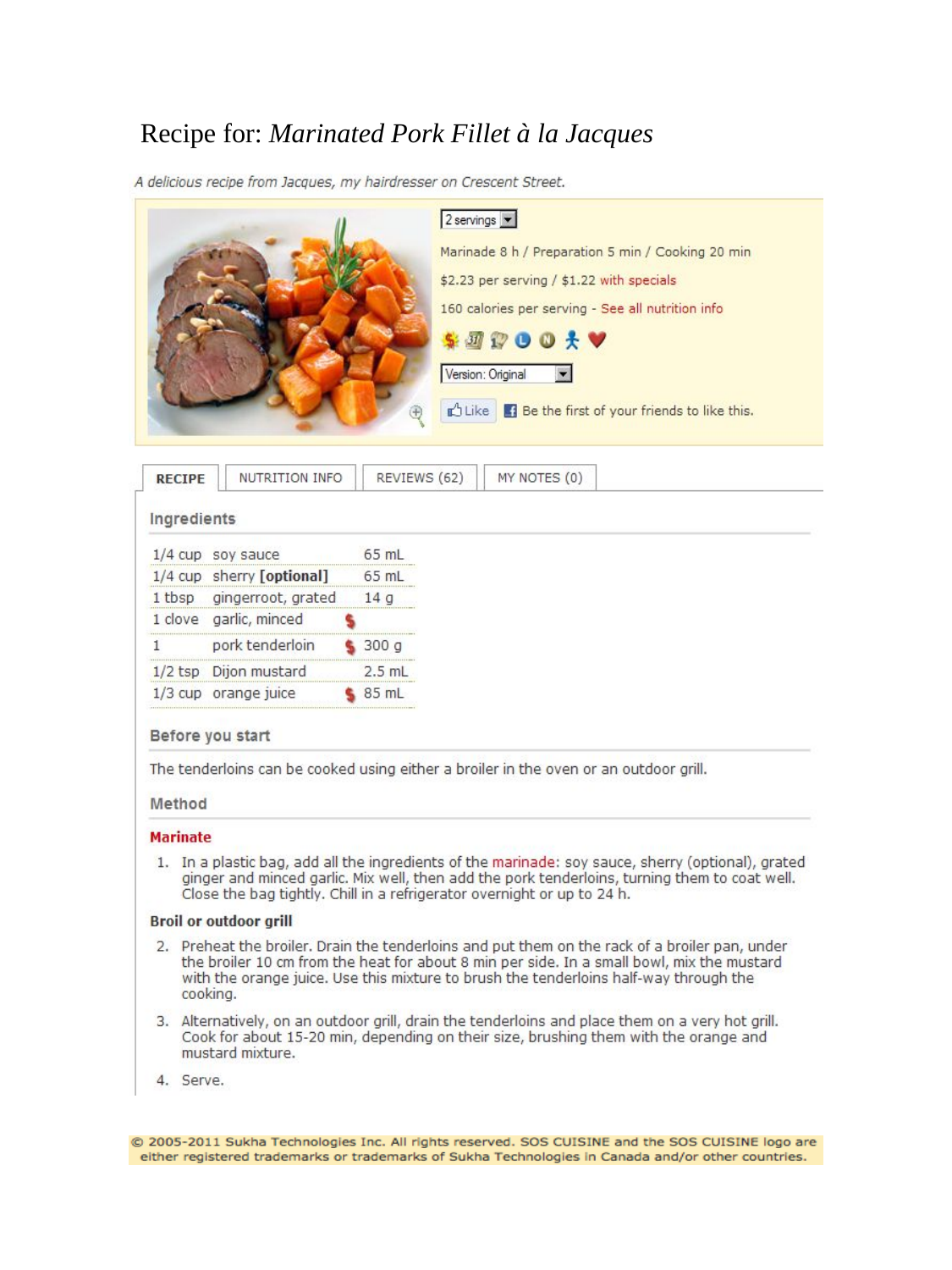

# Recipe for: Marinated Pork Fillet à la Jacques

## Slide 3
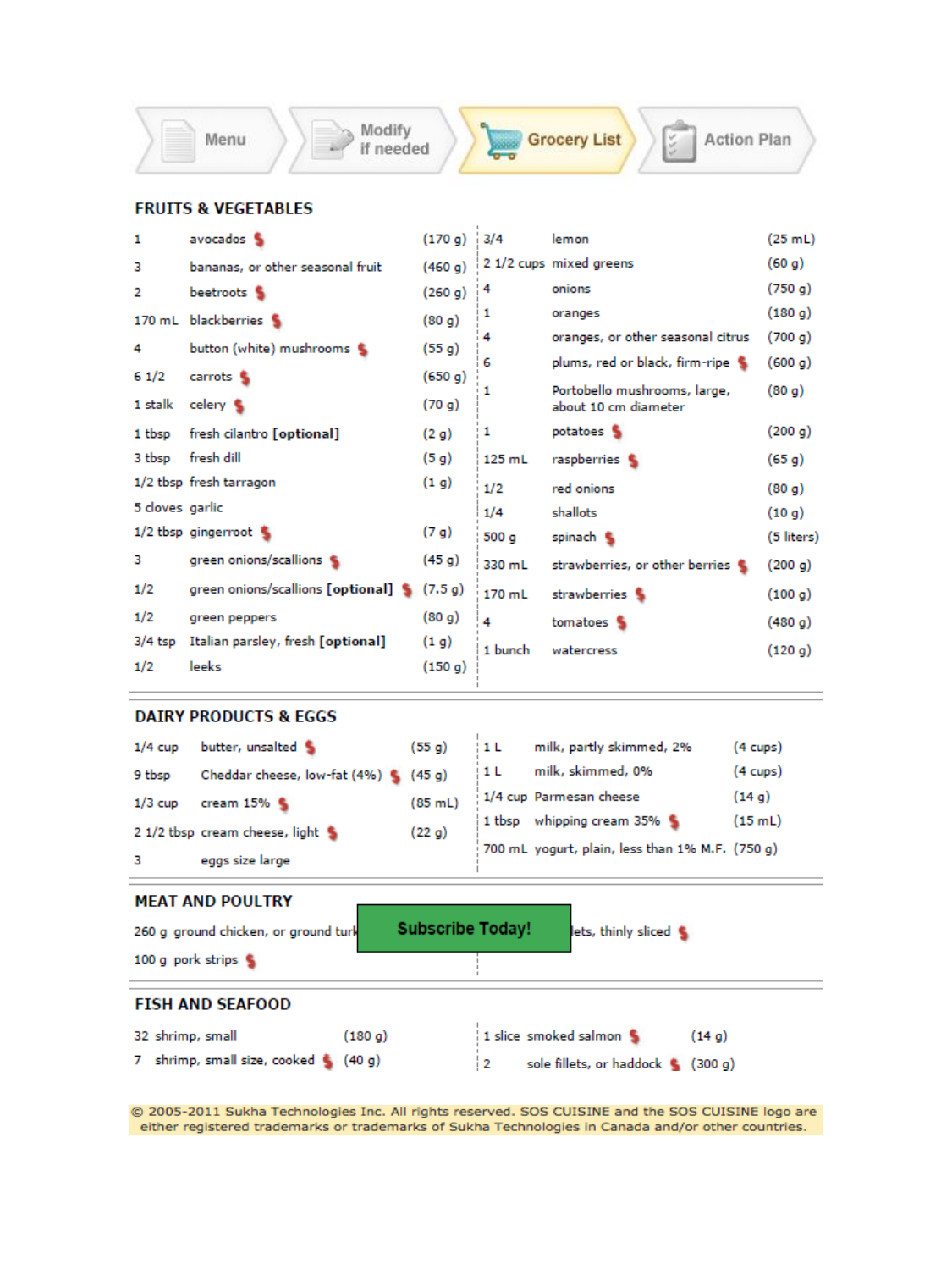

## Slide 4
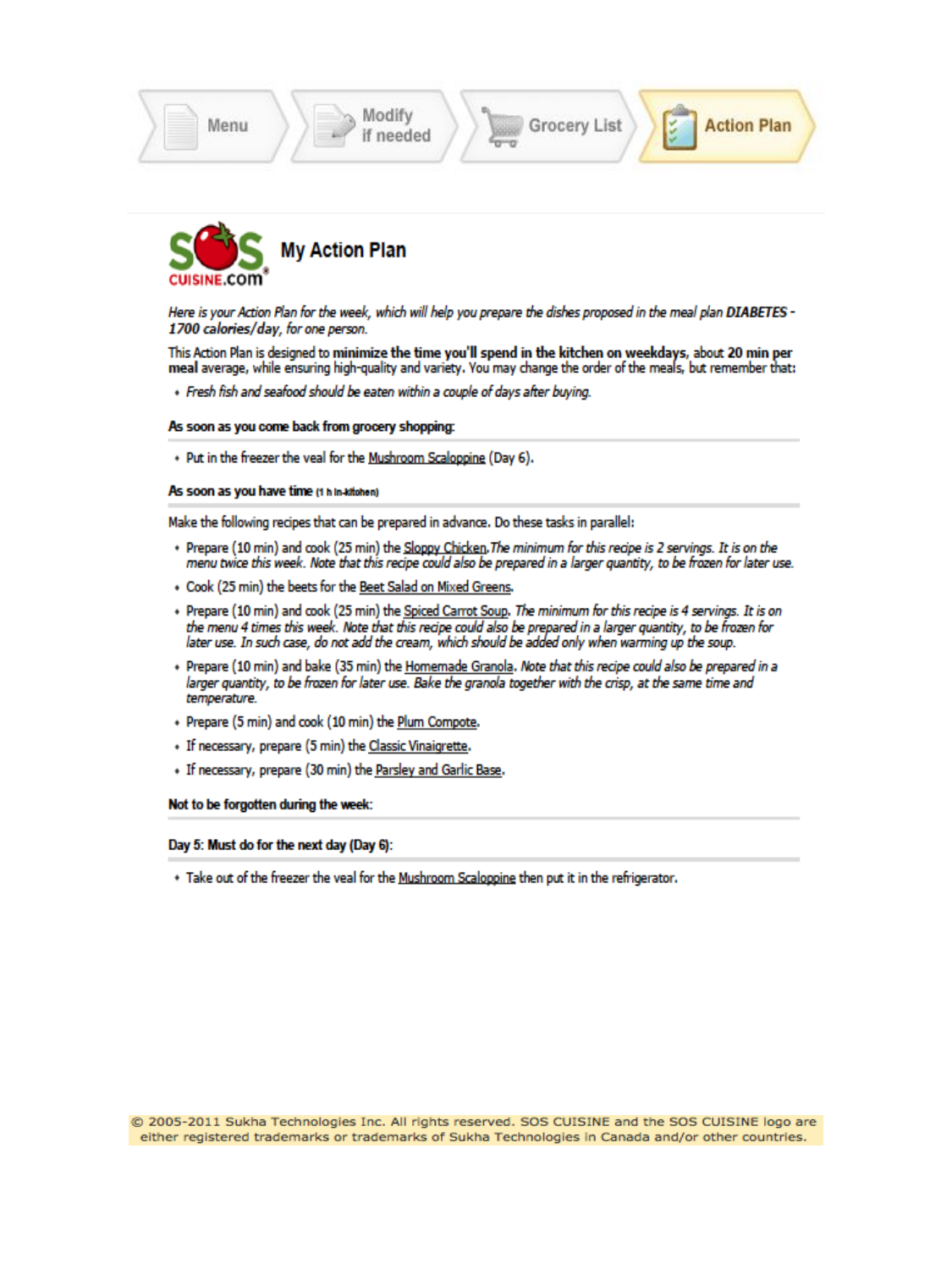

Supplement: Supplementary file 1 [file resprot_v2i2e41_app1.pptx]
